# Thickening of the pulmonary artery wall in acute intramural hematoma of the ascending aorta

**DOI:** 10.1186/1476-7120-5-1

**Published:** 2007-01-03

**Authors:** Martín Munín, María S Goerner, Martín Lombardero, Gustavo Sanchez, Juan C Pereira Redondo, Fernando Spernanzoni, Héctor Lardani, Víctor Torres

**Affiliations:** 1 Department of Echocardiography, CEMIC, Hospital Universitario Saavedra, Av. E. Galván 4102 Capital Federal Buenos Aires C1431FWO, Argentina

## Abstract

**Background:**

The occurrence of pulmonary artery obstruction in the course of acute aortic dissection is an unusual complication. The mechanism implicated is the rupture of the outer layer of the aorta and the subsequent hemorrhage into the adventitia of the pulmonary artery that causes its wall thickening and, at times, produces extrinsic obstruction of the vessel. There are no reports of this complication in acute intramural hematoma.

**Case presentation:**

An 87-year-old woman was admitted to the hospital in shock after having had severe chest pain followed by syncope. An urgent transesophageal echocardiogram revealed the presence of acute intramural hematoma, no evidence of aortic dissection, severe pericardial effusion with cardiac tamponade, and periaortic hematoma that involved the pulmonary artery generating circumferential wall thickening of its trunk and right branch with no evidence of flow obstruction. Urgent surgery was performed but the patient died in the operating room. The post mortem examination, in the operating room, confirmed that there was an extensive hematoma around the aorta and beneath the adventitial layer of the pulmonary artery, with no evidence of flow obstruction.

**Conclusion:**

This is the first time that this rare complication is reported in the scenario of acute intramural hematoma and with the transesophageal echocardiogram as the diagnostic tool.

## Background

Acute aortic dissection, acute intramural hematoma and aortic penetrating ulcer are entities with high mortality known as acute aortic syndromes. The usual fatal course of these conditions is closely related to their lethal complications [1]. The most frequent complications are rupture of the aorta with hemorrhage into the pericardial cavity, pleural space or mediastinum, or the occurrence of acute aortic valve regurgitation. The involvement of the pulmonary artery with wall thickening or even pulmonary flow obstruction in these circumstances is far less common. There are few reports in the literature of pulmonary artery involvement in the course of acute aortic dissection, but none of them in the scenario of acute intramural hematoma.

## Case report

**Figure 1:**
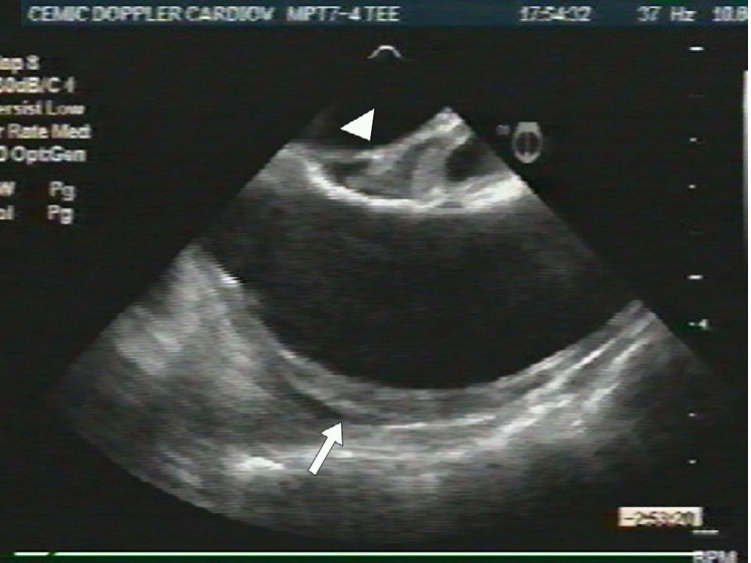
Transesophageal echocardiogram in the longitudinal plane showing aortic intramural hematoma compromising its anterior aspect (arrow). Notice the partially organized extravasated blood occupying the Theile sinus (arrowhead).

**Figure 2:**
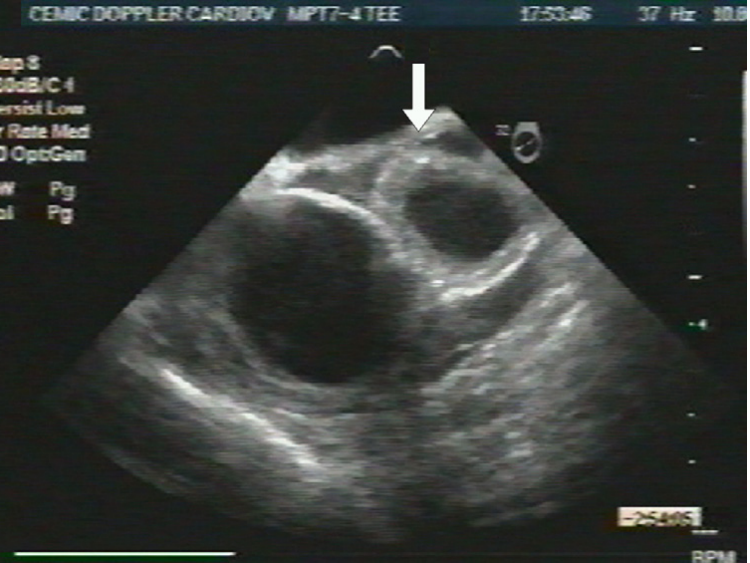
Short axis view at the level of the great vessels showing circumferential wall thickening of the pulmonary artery (arrow).

**Figure 3:**
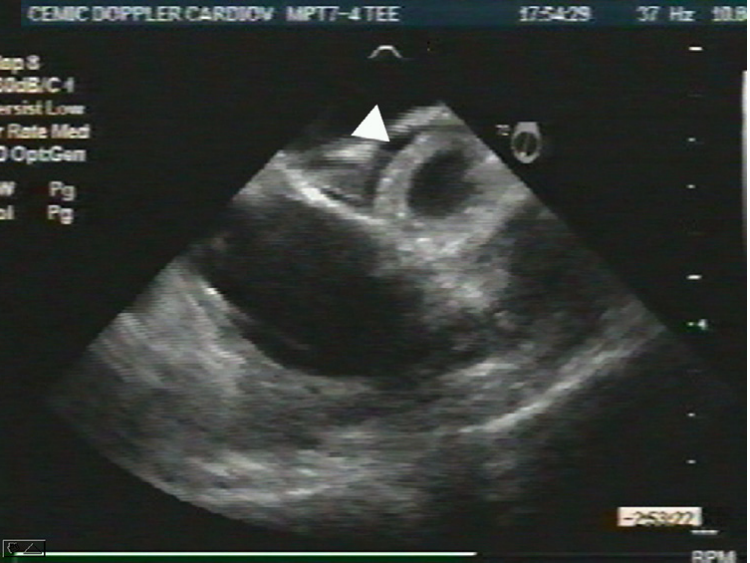
Long axis showing circumferential wall thickening of the right pulmonary artery (arrowhead).

**Figure 4:**
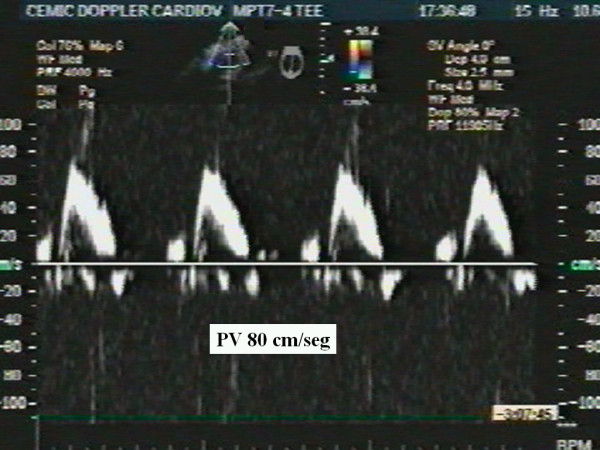
Evaluation with pulse wave Doppler of the right pulmonary artery showing normal flow velocity.

An 87-year-old woman with history of systemic hypertension and dyslipidemia was admitted to our hospital after having had severe chest pain of sudden onset radiating to the back and followed by syncope. She presented with marked hypotension (60/40), sinus tachycardia (130 beats per minute), and livid legs. She needed oral tracheal intubation, mechanical respiratory assistance, volume expansion and inotropic support. An urgent transesophageal echocardiogram revealed aortic intramural hematoma compromising the anterior wall of the ascending aorta (Fig. 1) with no evidence of aortic dissection, and severe pericardial effusion with cardiac tamponade and periaortic hematoma that involved the pulmonary artery generating circumferential wall thickening of its trunk (Fig 2) and right branch (Fig. 3) with no evidence of flow obstruction (Fig. 4) [see Additional file 1]. Urgent surgery was performed (drainage of pericardial hematoma and replacement of ascending aorta) but the patient died in the operating room.

The post mortem examination, in the operating room, confirmed that there was an extensive hematoma around the aorta and beneath the adventitial layer of the pulmonary artery with no evidence of flow obstruction.

## Conclusion

This rare complication of the acute aortic syndromes has been carefully described by Buja et al. in a case of aortic dissection [2]. Since the ascending aorta and pulmonary trunk share a common adventitia, the extravasation of blood into the adventitia of the ascending aorta may well extend into the adventitia of the pulmonary artery producing thickening of the artery wall and even causing, at times, compression of the pulmonary trunk or its main branches. Since extravasated blood in the adventitia is driven by aortic pressure, it can compress the lumen of pulmonary artery, which has relatively low pressure. The resultant hematoma in the adventitia around the pulmonary arteries plays an essential role in obstruction of these vessels, with no compromise of the medial layer. Given that it is located just behind the ascending aorta, obstruction often involves the right pulmonary artery [3].

There are few reports of this rare complication of acute aortic syndromes in the literature. Many of these reports are in patients who were initially thought to have pulmonary embolism. Indeed the ventilation-perfusion lung scan demonstrates mismatched absence of perfusion to the entire right lung in these cases [4,5]. Furthermore, a patient with this condition was treated with intravenous heparin and urokinase after being wrongly diagnosed with pulmonary embolism [6]. This rare complication should be kept in mind since the misdiagnosis as thromboembolism can lead to delayed and/or incorrect treatment in a particularly critical patient.

The reason we thought of presenting this case is threefold – there are few reports of this uncommon complication of acute aortic syndromes in the literature; this is the first time that it is described in the scenario of acute intramural aortic hematoma; and it is the first time that transesophageal echocardiogram is used as the diagnostic tool [7-9].

The transesophageal echocardiogram seems to be an accurate diagnostic tool not only to diagnose the aortic pathology but also to rule out this uncommon complication.

## Competing interests

The author(s) declare that they have no competing interests.

## Authors' contributions

MM carried out the transesophageal echocardiogram. MM and MSG performed review of the literature and wrote the paper. All authors read and approved of the final manuscript.

## Supplementary Material

Additional File 1 Video of the transesophageal echocardiogram showing an intramural hematoma compromising the anterior aspect of the ascending aorta with no evidence of aortic dissection. A circumferential wall thickening of the pulmonary artery can also be seen both in short and long axis view. There is no evidence of pulmonary flow obstruction as shown by color and pulsed wave Doppler. Click here for file
